# Spermidine in Colorectal cancer: friend or foe?

**DOI:** 10.1007/s00726-026-03554-w

**Published:** 2026-08-26

**Authors:** Sonia Coni, Rosa Bordone, Luca Campea, Shohei Ohno, Gianluca Canettieri, Enzo Agostinelli

**Affiliations:** 1 Department of Molecular Medicine, Sapienza University of Rome, Viale Regina Elena 291, Rome, 00161 Italy; 2 Department of Sensory Organs, Sapienza University of Rome, Rome, 00161 Italy; 3 International Polyamines Foundation ONLUS-ETS, Rome, 00159 Italy

**Keywords:** Polyamines, Spermidine, Colorectal cancer

## Abstract

Colorectal cancer (CRC) is a leading cause of cancer-related mortality, and its incidence is rising among individuals, especially young, underscoring the need for novel therapeutic strategies. CRC development is driven by genetic and epigenetic alterations in key oncogenic pathways and is accompanied by profound reprogramming of polyamine metabolism. In this context, spermidine (SPD), a central polyamine produced downstream of the MYC–ODC axis, is regarded as a pro-tumorigenic metabolite that sustains CRC growth by fueling eIF5A hypusination and MYC translation, thereby establishing a feed-forward MYC–ODC–polyamine–eIF5A circuit that promotes tumorigenesis. Recent studies also show that combined inhibition of polyamine biosynthesis and eIF5A hypusination synergistically impairs MYC translation and suppresses CRC growth in preclinical models, outperforming single-agent strategies such as ornithine decarboxylase (ODC) blockade with difluoromethylornithine (DFMO) and highlighting the translational potential of dual targeting of this axis. Conversely, other lines of evidence indicate that excessive SPD or altered flux through the polyamine pathway can be cytotoxic: multiple reports demonstrate that high-dose SPD triggers apoptosis, while experimental models suggest that SPD and epithelial eIF5A hypusination can protect against colitis and colitis-associated cancer by supporting the translation of detoxifying enzymes. Overall, the literature points to a dual role of SPD, and the aim of this review is to dissect the functions of SPD in CRC, to summarize and critically discuss the potential benefits of this polyamine, and to evaluate how it might be rationally exploited as a therapeutic agent in cancer.

## Colorectal cancer and polyamines

Colorectal cancer (CRC) is currently the third leading cause of cancer related death worldwide, with approximately one million deaths per year (Sung et al. 2021), and represents a major healthcare challenge due to the need for effective screening and treatment strategies.

Colorectal cancer predominantly affects individuals over fifty years of age. However, in recent years a worrisome increase in CRC incidence among individuals younger than fifty has been observed. Although the underlying causes of early onset CRC are not fully understood, a realistic correlation has been proposed between this condition and a proinflammatory diet characterized by high intake of sweet beverages and a high Western diet score, rich in processed meat, saturated fats and sugars, and poor in fruit and vegetables (Ivy et al. 2025). Several factors increase the risk of developing CRC, including age (Maffei et al. 2016), family history of cancer and hereditary syndromes (Stigliano 2014), smoking (Chen et al. 2015), diet and gut microbiota composition (Park et al. 2005), lack of physical activity, chronic inflammation such as colitis and inflammatory bowel disease (IBD) (Dyson 2012; Yashiro 2014), and persistent viral infections leading to chronic inflammation (Nayudu 2012).

These considerations highlight the urgent need to develop novel therapeutic strategies, which in turn requires a deeper understanding of the molecular mechanisms underlying the pathogenesis of this tumor. Colorectal cancer is a multifactorial and multistep neoplasm that arises through the accumulation of specific genetic alterations and dysregulation of oncogenic pathways, including WNT, KRAS, PI3K and TGFβ (Arruabarrena-Aristorena et al. 2018).

Several therapeutic options are currently available: surgery for early stage disease; chemotherapy regimens such as FOLFOX, FOLFIRI and 5-fluorouracil; radiotherapy; targeted therapies, including EGFR inhibitors (e.g. cetuximab) and VEGFR inhibitors (e.g. bevacizumab) (Częścik et al. 2026). Immunotherapy with immune-checkpoint inhibitors is a relevant therapeutic approach for tumors belonging to the subclass characterized by high microsatellite instability (MSI-high) and defective mismatch repair (dMMR) (Le et al. 2015), (Sahin et al. 2019), (Lemery et al. 2017).

Despite this knowledge, metastatic CRC remains essentially incurable, and resistance to targeted therapies frequently emerges, often through mechanisms that restore signaling pathway activity at downstream levels.

Therefore, alternative approaches aimed at identifying and targeting downstream effectors of oncogenic pathways are needed.

In this context, several studies have demonstrated that polyamine metabolism represents a relevant actionable tumorigenic driver in CRC (Gerner and Meyskens 2004), (Coni et al., 2020), (Coni et al. 2023) and that CRC cells exhibit increased content of polyamines as well as elevated level of ODC, the first rate-limiting enzyme in polyamine production (Hu 2005). Polyamines are small polycationic molecules involved in multiple cellular functions, including cell survival and proliferation, and their intracellular levels are frequently increased in several cancers, including colorectal tumors.

**Fig. 1:**
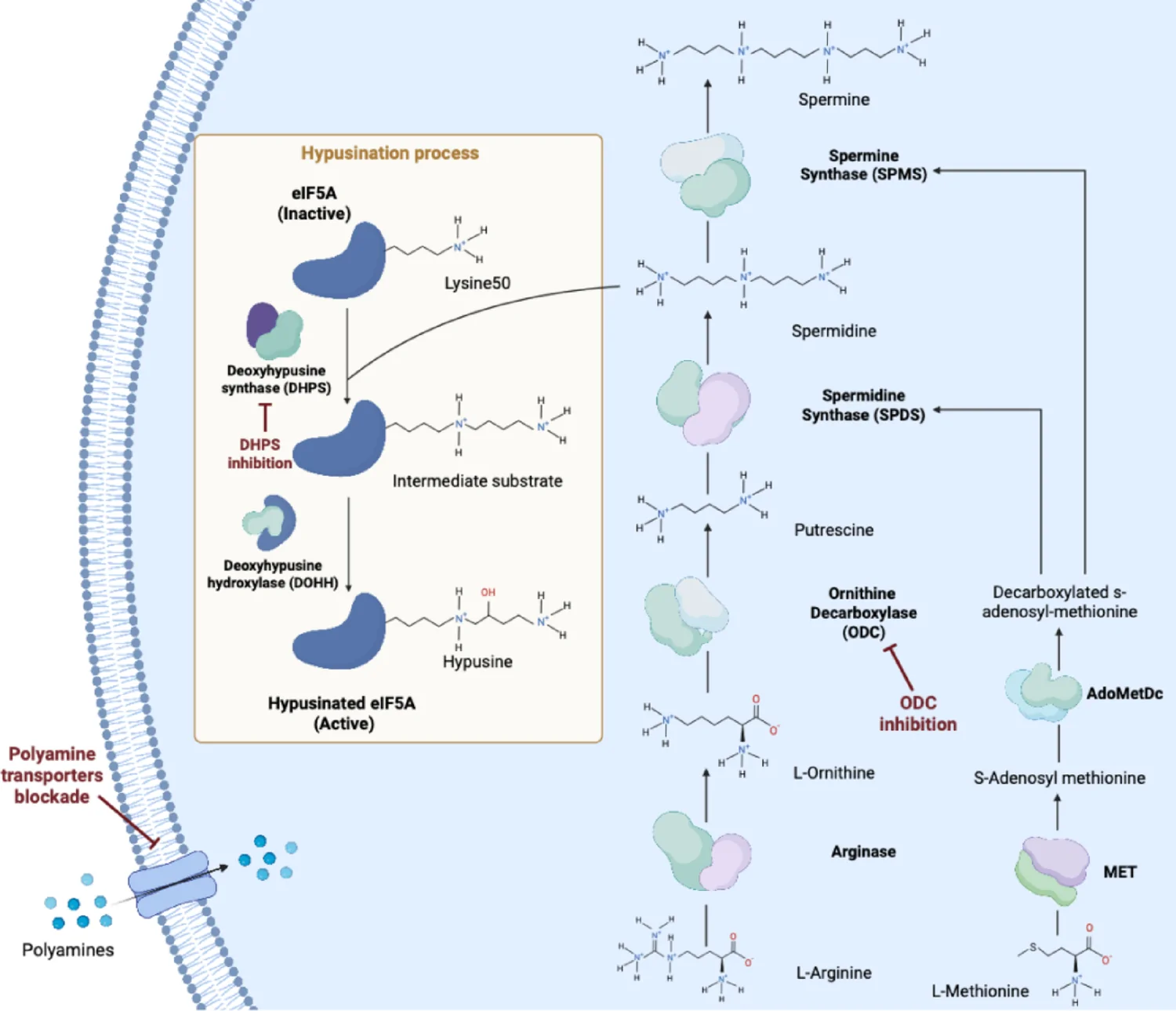
Polyamine metabolism and eIF5A hypusination pathway. The scheme illustrates the metabolic flux of the three primary polyamines: putrescine, spermidine, and spermine. The pathway begins with the conversion of arginine (via ornithine), followed by a series of sequential enzymatic steps that regulate polyamine homeostasis. A critical downstream effector of this pathway is the post-translational modification of the eukaryotic translation factor eIF5A. This process, known as hypusination, utilizes a butylamine moiety derived from spermidine to activate eIF5A. Once hypusinated, eIF5A facilitates the ribosomal elongation of specific target mRNAs, particularly those containing difficult-to-translate sequences such as polyproline motifs

Polyamine biosynthesis begins with the decarboxylation of ornithine to putrescine (PUT), a reaction catalyzed by the enzyme ODC (Fig. 1). Putrescine is then converted into Spermidine (SPD) by spermidine synthase (SRM), and SPD is further converted into spermine (SPM) by spermine synthase (SMS) (Casero et al. 2018).

A key role in controlling the rate of polyamine production is played by ODC, which is tightly regulated at both the transcriptional and posttranscriptional levels. Notably, ODC is a direct transcriptional target of the MYC oncogene, a major driver of colorectal tumorigenesis (Bello-Fernandez et al. 1993). Because of its intrinsically disordered, “flat” protein structure, MYC is widely regarded as an undruggable target, and no effective therapeutic strategies directly inhibiting MYC are currently available (Bordone et al. 2024); Dang et al. 2017)

Consequently, targeting downstream oncogenic MYC-regulated pathways, such as the polyamine/hypusine axis, has emerged as an attractive alternative approach (Coni et al., 2020).

Hypusination is a unique posttranslational modification that results from the covalent addition of a SPD moiety to lysine 50 of the translation factor eIF5A, through the sequential actions of deoxyhypusine synthase (DHPS) and deoxyhypusine hydroxylase (DOHH).

eIF5A, is the only known hypusinated protein and it functions as a regulator of translational initiation and/or elongation (Gutierrez et al., 2013). Among the eIF5A translational targets there is the MYC transcription factor itself, which generates a feedback loop, whereby MYC promotes polyamine synthesis and is itself induced at the translational level by the polyamine/hypusine pathway (Coni et al., 2020). This molecular axis can be efficiently targeted by drugs inhibiting enzymes involved in the conjugation of SPD on eIF5A (i.e. DHPS) or through inhibition of polyamine production, starting from ODC activity. Furthermore, since the intracellular polyamine levels can be also regulated by import through transporters or endocytic mechanisms (Hamouda et al. 2021), (Van Veen et al. 2020) the inhibition of polyamine transporters can be also exploited to downregulate intracellular polyamine levels.

Among all polyamines, SPD stands out as the most versatile and has recently attracted considerable research interest due to its multifaceted biological roles.

Indeed, SPD can function either as a promoter of cell survival and tumorigenesis or as a cytoprotective agent that enhances cellular resilience against death stimuli, or as inducer of apoptosis (Fan et al. 2020), (Seiler and Raul 2005), (Tahara et al. 2025).

In this review we are going to discuss the different contribution of SPD in preclinical models of CRC and the most recent scientific advances about CRC therapeutic opportunities by balancing the intracellular content of the polyamine SPD. 

## The dual role of spermidine in Colorectal tumorigenesis: in vivo evidence

Several works have demonstrated that SPD is crucial for cellular homeostasis through electrostatic interactions stabilizing DNA/RNA, promoting protein translation, and modulating ion channels (Igarashi and Kashiwagi 2010) (Dever and Ivanov 2018).

In terms of cellular responses, it has been documented the ability of SPD to induce autophagy, reduce oxidative stress and inflammation, and support mitochondrial function (Hofer et al. 2024), (Vrijsen et al. 2020). Since these processes decline with age, along with the tissue concentration of SPD, this polyamine is considered a potential longevity molecule. (Minois 2014).

**Fig. 2:**
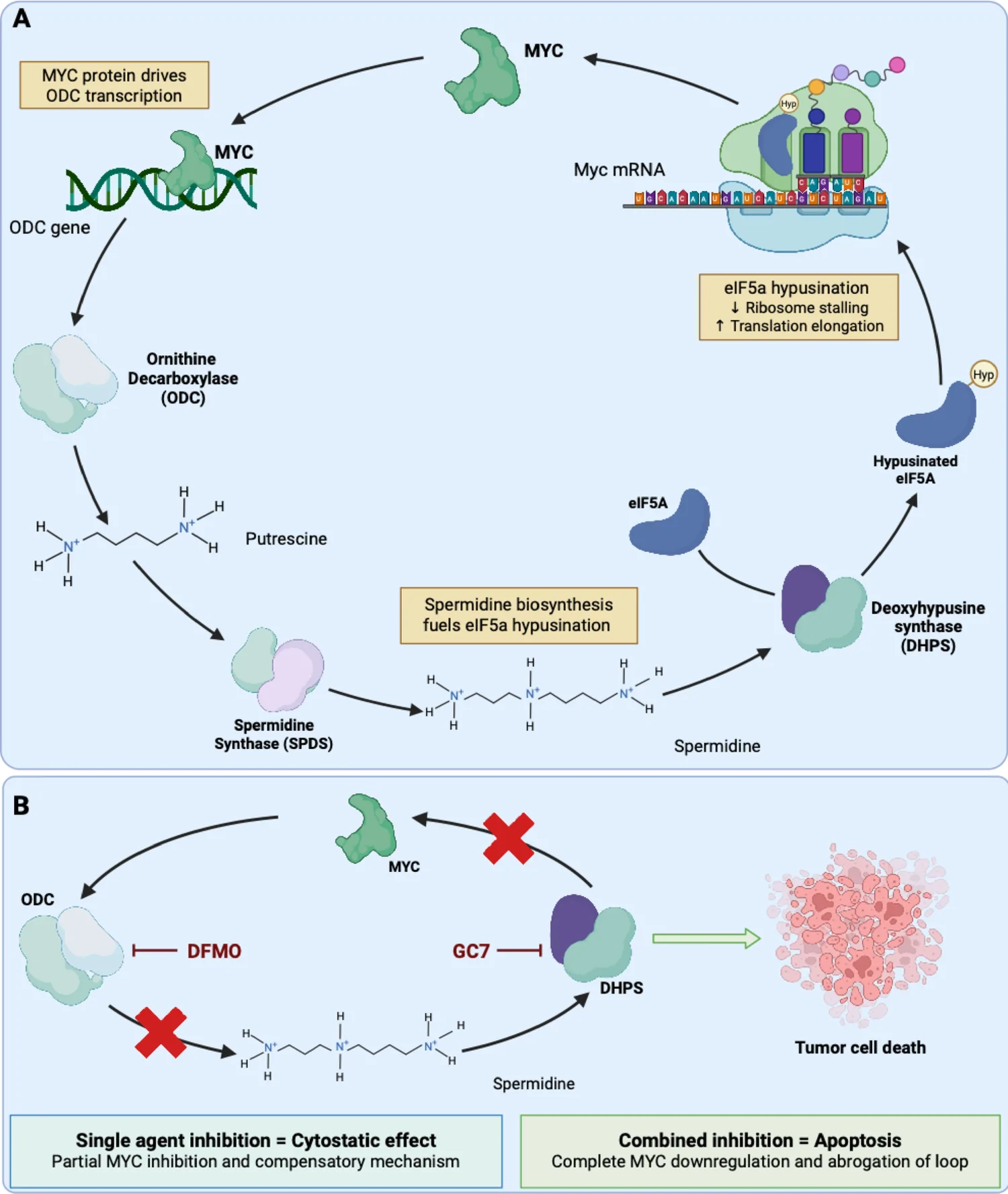
Therapeutic disruption of the polyamine-MYC feed-forward loop in colorectal cancer. (A) Elevated polyamine levels facilitate the hypusination of eIF5A, which promotes the translational elongation of the MYC oncogene. MYC subsequently drives the transcription of ornithine decarboxylase (ODC), the rate-limiting enzyme in polyamine biosynthesis, further fueling the cycle. (B) Pharmacological intervention targeting this axis involves the inhibition of ODC by difluoromethylornithine (DFMO) or the blockade of deoxyhypusine synthase (DHPS) via GC7. While monotherapy with either agent induces cytostatic effects and growth arrest, the synergistic combination of DFMO and GC7 triggers robust apoptotic cell death, effectively counteracting CRC progression

In CRC, spermidine biosynthesis is upregulated via the MYC-ODC axis, fueling tumorigenesis through eIF5A hypusination, which enhances MYC translation (Coni et al., 2020)(Fig. 2). Human cells express two eIF5A isoforms: constitutive eIF5A1 (overexpressed in proliferating and in cancer cells) and tissue-specific eIF5A2 (amplified in ovarian and CRC malignancies, driving cellular transformation) (Caraglia et al. 2013). Hypusinated eIF5A resolves ribosome stalling at polyproline motifs, enabling peptide bond formation and translation elongation (Schmidt et al. 2016). Without eIF5A, ribosomes stall at these sites, disrupting oncogene synthesis. Recent evidence shows that inhibition of DHPS restricts CRC growth and tumorigenesis in mouse models by preventing eIF5A-dependent translation elongation of MYC at five specific Coding DNA Sequence (CDS) pausing sites, thereby uncovering a MYC–ODC–polyamine feedback loop that can be therapeutically targeted (Coni et al., 2020). Clinical trials using single agents targeting the polyamine pathway have generally proven to be disappointing (Casero et al. 2018), except for DFMO, an irreversible inhibitor of ODC, which shows success in the treatment of anaplastic gliomas (Levin et al. 2018), in ongoing clinical trials for neuroblastoma (Hogarty et al. 2024) and in prevention of CRC by combination with sulindac (Raj et al. 2013). Nevertheless, DFMO treatment is generally thought to exert a cytostatic, rather than a cytotoxic effect, mainly due to the activation of compensatory mechanisms that result in increased polyamine transport or upregulation of SAMDC (S-adenosylmethionine decarboxylase encoded by *AMD1* gene) which catalyzes the decarboxylation of S-adenosylmethionine to form decarboxylated S-adenosylmethionine (dcAdoMet), that serves as an aminopropyl donor to generate SPD (Casero et al. 2018). Other studies from (Coni et al. 2023), revealed that the combined targeting of polyamine biosynthesis and eIF5A hypusination is effective in counteracting CRC growth (Fig. 2).

In contrast with individual inhibitions, simultaneous inactivation of ODC (with DFMO) and DHPS (with GC7, N1-guanyl-1,7-diaminoheptane) results in a synergistic inhibition of tumor growth and induces apoptosis in CRC cells, and in APC^Min/+^ mice. This phenomenon was also observed in other cancer types such as neuroblastoma and endometrial carcinoma (Kim et al. 2022), (Schultz et al. 2018). The described effect was likely due to the different intracellular levels of SPD achieved. Indeed, DFMO administration is insufficient to completely inactivate eIF5A, as well as the GC7 treatment alone. Conversely, the two combined treatments lead to a complete downregulation of MYC levels, due to the blockage of eIF5A hypusination on one side, and to the limited ribosomal assembly, because of the reduction of intracellular polyamine pool following DFMO-mediated ODC blockage. Then, treatments with DFMO and GC7 alone achieve the partial inhibition of MYC causing cell cycle arrest, while the dual treatment, targeting simultaneously ODC and DHPS completely abrogates the intracellular content of MYC oncogene, leading to cell death. Similarly, Guo et al. (Guo et al. 2020) demonstrated that combined inhibition of spermine synthase (SMS) and MYC signaling synergistically induces apoptosis and tumor regression in CRC models, with a mechanism linked to the upregulation of the proapoptotic protein Bim (BCL2L11, a Proapoptotic Protein). Genetic SMS deletion reprograms polyamine metabolism, resulting in SPD accumulation that sensitizes CRC cells to the bromodomain inhibitor JQ1 (a thienotriazolodiazepine), thereby enhancing apoptosis and promoting tumor regression. Specifically, SMS suppresses Bim transcription by counteracting hdac-mediated inhibition of FOXO3a acetylation, whereas MYC downregulates Bim post-transcriptionally via induction of the micro RNAs miR-19a/19b. Individual pathway inhibition fails to restore Bim levels, but dual blockade robustly upregulates Bim and triggers apoptosis.

MYC regulates polyamine synthesis at multiple levels by inducing key biosynthetic genes, including *ODC*, *SRM (spermidine synthase)* and *AMD1*, thereby increasing SPD levels (Bello-Fernandez et al. 1993), (Fernandez et al. 2003), (Forshell et al. 2010). Spermidine, in turn, supports MYC expression, cell proliferation, and malignant transformation (Tabib and Bachrach 1999) .

In apparent contrast with the increased content of SPD during intestinal tumorigenesis and its reported pro-tumorigenic role, other evidence highlights a protective effect of exogenous SPD supplementation in mouse models of colitis associated cancer. Gobert et al. showed that *SMOX* (spermine oxidase) gene deletion, which catalyzes SPD production from SPM, reduces colonic SPD levels and exacerbates dextran sodium sulfate (DSS) induced colitis compared with wild-type mice (Gobert et al. 2022). Colonic SPD is inversely correlated with histopathological severity in wild-type mice. Oral SPD supplementation protected against DSS colitis and azoxymethane AOM/DSS-induced tumorigenesis, associated with reduced expression of α-defensins, key innate immune effectors in neutrophils and Paneth cells, and preservation of a protective gut microbiota. Notably, endogenous SMOX levels are decreased in inflammatory bowel disease (IBD) patients and colitis-associated high-grade dysplasia, suggesting that SPD supplementation is a potential therapeutic strategy. The same research group further reported the protective effects of spermidine on the intestinal epithelium during infection with *Citrobacter rodentium*, a murine model of enteropathogenic *Escherichia coli* (EPEC) infection (Gobert et al. 2024). Spermidine promotes eIF5A hypusination, which regulates translation of enzymes involved in detoxifying deleterious aldehydes in the infected mucosa. *Dhps* deletion worsened clinical, histological, and proinflammatory outcomes in infected mice, suggesting that epithelial hypusination mitigates infectious colitis. Impaired hypusination is observed in ulcerative colitis, Crohn’s disease samples, and patient-derived organoids, as well as in *Dhps* conditional colonic knockout mice, which spontaneously develop colonic hyperplasia, epithelial proliferation, crypt distortion, and inflammation, with exacerbated chemical carcinogenesis (Gobert et al. 2023). This phenotype stems from increased aldehyde adducts due to defective translation of detoxifying enzymes, including glutathione S-transferases and aldehyde dehydrogenases. A similar mechanism operates in *Apc*-deletion models of CRC, where inducible *Dhps* knockout in colonic epithelia accelerates tumorigenesis (Gobert et al. 2025). In this model, epithelial hypusination prevents colitis and CRC initiation, and SPD supplementation could enhance this pathway therapeutically.

This apparent eIF5A paradox likely reflects a context-dependent role, where hypusination prevents tumorigenesis by maintaining mitochondrial homeostasis and mucosal integrity, yet promotes established tumor growth by fueling the high translational demands of cancer cells, making its inhibition effective only in established malignancy.

Finally, it needs to be mentioned the role of SPD within the tumor microenvironment (TME), where its level is critically regulated and contrasting information are available in literature. Tumor cell metabolism plays a key role in driving immune evasion, and polyamine pathways have been found to be closely related to cancer cells. However, the mechanism of crosstalk between polyamines and TME remains unclear. Polyamines are known to encourage a tumor-permissive microenvironment trough different mechanisms (Dryja et al. 2021), (Zhang et al. 2023). High expression of polyamine related genes was associated with aggressive molecular phenotypes, poor prognosis, diminished antitumor immunity and poor response to immunotherapy (Zhang et al. 2023). Immunologically cold cancers often exhibit extremely high levels of polyamine metabolism. Depletion of polyamines from immunosuppressive tumor microenvironments can reprogram the microenvironment to a more immunological permissive phenotype (Holbert et al. 2024). Other observations in literature suggest an antitumoral role of SPD in the TME. An interesting work by Miao H. et al., discuss the activity of the tumor-infiltrating macrophages highly expressing ABHD5 (α/β-hydrolase domain-containing 5) an enzyme involved in lipolysis, which suppress spermidine production by inhibiting the SRM expression, thereby promoting CRC growth by reducing reactive oxygen species (ROS) accumulation and impairing SPD synthesis (Miao et al. 2016). In this direction, targeting ABHD5-high macrophages to boost SPD may inhibit tumor progression, underlining a SPD antitumor role in the TME.

**Fig. 3:**
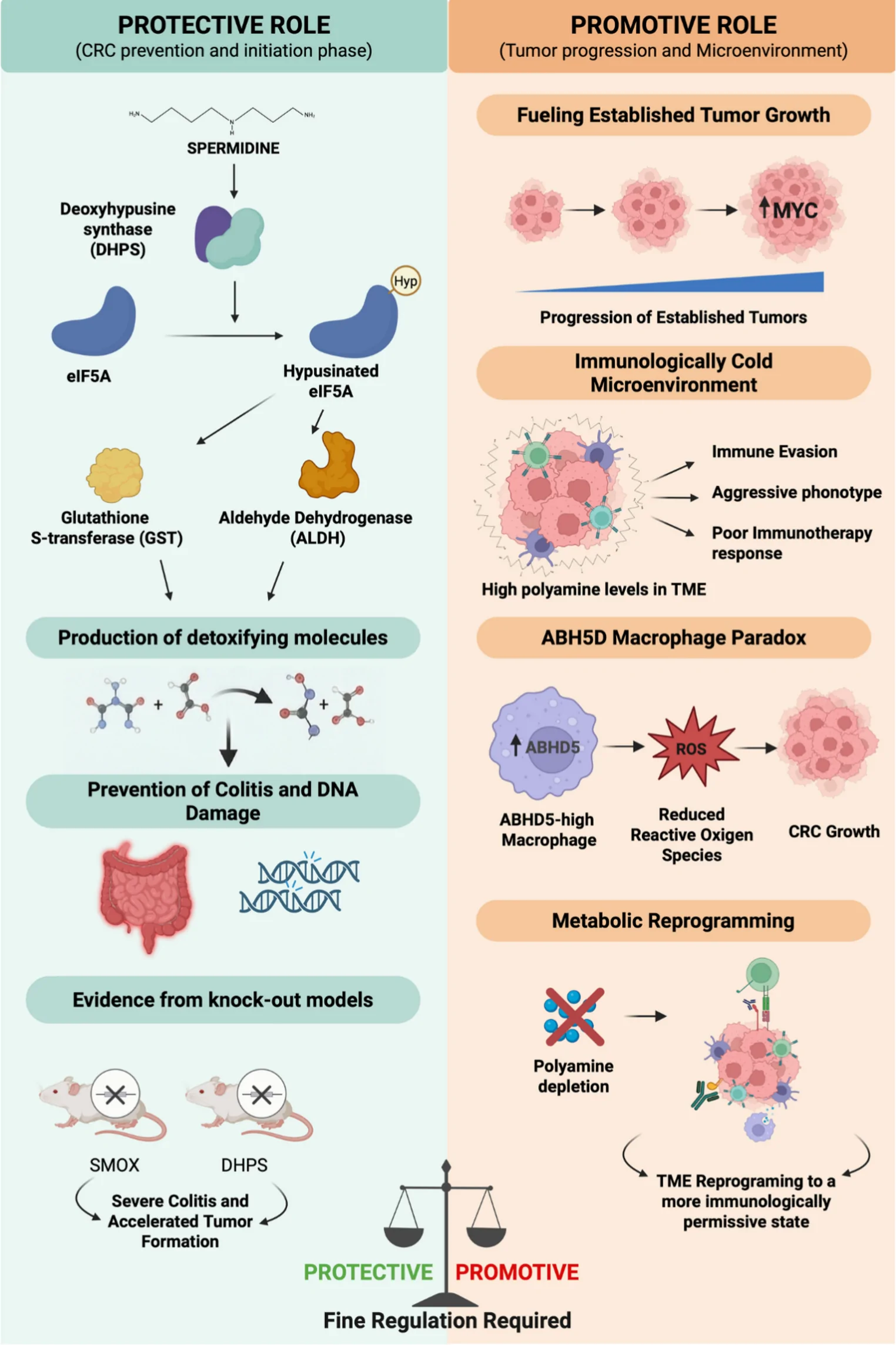
Dual role of the polyamine-eIF5A axis in Colorectal Cancer (CRC) pathogenesis and therapy. Under physiological conditions, spermidine-dependent eIF5A hypusination is essential for the efficient translation of detoxifying enzymes, including GST and ALDH. This enzymatic activity prevents colitis, limits DNA damage, and suppresses tumor initiation. The critical nature of this cytoprotective mechanism is evidenced by knockout models (Smox^-/-^ and Dhps^-/-^), where the loss of polyamine signaling leads to severe colitis and accelerated tumorigenesis. In established tumors, the pathway is exploited to fuel rapid proliferation of transformed cells via MYC translation. High polyamine concentrations within the tumor microenvironment (TME) contribute to an immunologically cold state, characterized by immune evasion and resistance to immunotherapy. Depleting polyamines can reprogram the TME into an immunologically permissive state, enhancing anti-tumor immune infiltration and potentially sensitizing tumors to therapeutic intervention

In conclusion, SPD functions as a complex metabolic rheostat in intestinal tumorigenesis, exerting a dual role that spans from protecting mucosal integrity and priming anti-tumor immunity to fueling neoplastic progression and TME-driven immunosuppression, depending on the specific cellular context and the stage of disease (Fig. 3).

## The multifaceted role of spermidine in CRC culture models

In addition to the conflicting results reported in various animal models, the literature on CRC cell lines has historically presented a fragmented landscape regarding the response to exogenous SPD treatment. Discrepant outcomes, ranging from enhanced cell survival to induced cytotoxicity, have been documented across several decades of research (Sugiura et al. 1984) (Averill-Bates et al., 1993), (Sharmin et al. 2001), (Calcabrini et al. 2002) (Pietrocola et al. 2015), (Wang et al. 2018); Holbert et al. 2020).These inconsistencies often stemmed from variations in experimental conditions, such as polyamine bioavailability and the presence of bovine serum amine oxidases in the culture media. To systematically address these discrepancies, Tahara et al. recently employed a refined experimental model using polyamine-depleted CRC cells, allowing for a precise evaluation of SPD effects across a broad concentration gradient (Tahara et al. 2025). Their findings reveal a clear dose-dependent, dualistic behavior of SPD. At low, physiologically relevant concentrations (1–10 µM), SPD acts as a pro-tumorigenic factor by promoting cell proliferation. This effect is mechanistically driven by the induction of eIF5A hypusination, which in turn facilitates the efficient translation of MYC, a master regulator of oncogenic growth. Conversely, when the concentration of SPD reaches higher thresholds (> 50–100 µM), the response shifts toward a marked cytotoxicity, primarily mediated by the generation of ROS.

Furthermore, the study provides insights into the complex relationship between SPD and autophagy, a process often misconstrued in earlier reports. While it is well-established that SPD can modulate autophagy through the eIF5A-mediated translation of key autophagic regulators, Tahara et al. demonstrated that high doses of SPD induce only a transient inhibition of the autophagic flux. This temporary blockade is not a direct signaling effect of the polyamine itself, but rather a stress-induced response to the oxidative damage caused by the oxidative deamination of SPD by Bovine Serum Amine Oxidase (BSAO), a copper enzyme typically contained in fetal bovine serum, in accordance with the observation made by Holbert CE et al. (Holbert et al. 2020). Importantly, in the paper of Tahara et al. SPD administration at low concentrations in CRC cells, promotes eIF5A hypusination without altering the autophagic flux at any time point, suggesting that its pro-proliferative activity is independent of autophagy modulation.

A particularly relevant contribution of the work carried out by Tahara et al. is the biochemical characterization of N1-guanyl-1,7-diaminoheptane (GC7). While GC7 is globally recognized as a potent and specific inhibitor of DHPS - the enzyme responsible for the first step of eIF5A activation - this study is the first to describe its secondary enzymatic activity as an inhibitor of BSAO. This discovery is highly relevant in the polyamine field, as it redefines the role of GC7: it can simultaneously prevent the pro-tumorigenic activation of eIF5A (via DHPS inhibition) and protect cells from the artefactual oxidative stress induced by SPD oxidation in vitro (via BSAO inhibition). This dual-target mechanism provides a much-needed explanation for the opposing effects of SPD doses and establishes a more rigorous framework for future pharmacological interventions targeting the polyamine pathway.

## Conclusion and future perspective

Polyamine metabolism has emerged as a relevant component of redox homeostasis in colorectal cancer (CRC), with spermidine occupying a context-dependent position at the interface between tumor promotion and tumor suppression (Guo et al. 2020) (Casero et al. 2018). In CRC and related intestinal models, dysregulated polyamine catabolism can contribute to oxidative stress through the production of hydrogen peroxide, that can form, by Fenton reaction, the highly toxic hydroxyl radicals, as the singlet oxygen (^1^O^2^), the radical superoxide anion (O^2.−^) and the hydroxyl radical (HO.) (Agostinelli and Seiler 2006), particularly when spermine oxidase or spermidine/spermine N1-acetyltransferase pathways are activated (Casero and Pegg 2009). At the same time, spermidine has also been associated with anticancer effects, including autophagy induction, mitochondrial protection, and suppression of oxidative damage in several experimental systems, suggesting that its biological outcome depends strongly on dose, cellular context, and metabolic state (Al-Habsi et al. 2022) (Zimmermann et al. 2023). Then, the complex landscape of polyamine research increasingly points toward a dualistic role for SPD, where its biological impact is strictly governed by the metabolic context and the specific enzymatic landscape of the tissue. While it has long been recognized that the in vitro cytotoxicity of SPD is largely an indirect effect - driven by its oxidative deamination into reactive products, aldehydes and hydrogen peroxide, by BSAO - recent studies, such as the one by Tahara et al., have been pivotal in reconciling these long-standing discrepancies. By decoupling the eIF5A-mediated proliferative signaling from the oxidase-mediated cell death, these reports provide a rigorous framework to translate “laboratory artifacts” into potential therapeutic strategies.

The identification of GC7 as a dual inhibitor of both DHPS and BSAO in vitro represents a significant milestone in the polyamine field. This discovery not only necessitates a critical re-evaluation of decades of in vitro data where GC7 was mainly used as a “specific” hypusination inhibitor, but also highlights a sophisticated pharmacological opportunity. Future research should investigate the feasibility of using bifunctional molecules or combination therapies that can selectively modulate either the hypusination axis (to curb MYC-driven proliferation) or the oxidative axis (to induce tumor-specific apoptosis).

A primary challenge remains the translation of these observations from the bench to the bedside. The demonstration that GC7 acts as a dual inhibitor of both DHPS and BSAO is a double-edged sword: while it demands extreme caution in interpreting past and future in vitro data, it also suggests that GC7 could be utilized as a precision tool to decouple the pro-survival effects of eIF5A hypusination from the oxidative stress responses. Future research should prioritize determining whether an equivalent “oxidative bypass” exists in vivo. Specifically, it is imperative to investigate if human endogenous oxidases, such as SSAO/VAP-1, can be “primed” to locally oxidize SPD within the tumor microenvironment. If confirmed, this would transform SPD from a simple metabolite into a locally activatable pro-drug.

In this context, the targeted delivery of high-dose SPD via nanoparticle-based formulations represents a promising frontier. By exploiting the high expression of amine oxidases in specific tumor types or their vascular stroma, we could trigger a localized “oxidative burst” - generating toxic aldehydes directly in the niche where cancer cells reside (Tabor et al. 1964), (Averill-Bates et al. 2005),(Averill-Bates et al. 2008). This approach would shift the therapeutic focus from systemic polyamine depletion to a localized, oxidase-mediated chemo-ablation.

Furthermore, the integration of SPD treatment with therapies that impair cellular redox defenses (such as glutathione peroxidase inhibitors) could synergistically enhance this localized cytotoxicity.

Simultaneously, at the opposite end of the spectrum, the pro-tumorigenic axis (SPD-eIF5A-MYC) identified at low doses highlights a critical risk: the gut microbiota, a major reservoir of systemic polyamines, may inadvertently fuel CRC progression by maintaining sub-toxic, proliferative SPD levels. Deciphering the crosstalk between microbial SPD production, host amine oxidase activity, and mucosal eIF5A hypusination will be essential to understanding the systemic regulation of CRC and to developing personalized nutritional or pharmacological interventions.

Ultimately, the work of Tahara et al. does not merely resolve a laboratory discrepancy; it provides the biochemical blueprint for a new generation of polyamine-targeted therapies that leverage the precise control of eIF5A activity and the strategic exploitation of oxidative metabolism.

## Data Availability

No datasets were generated or analysed during the current study.

## Abbreviations

CRC: Colorectal cancer
SPD: Spermidine
DFMO: Difluoromethylornithine
ODC: Ornithine decarboxylase
PUT: Putrescine
SRM: Spermidine synthase
SPM: Spermine
SMS: Spermine synthase
DHPS: Deoxyhypusine synthase
DOHH: Deoxyhypusine hydroxylase
SAMDC: S-adenosylmethionine decarboxylase
dcAdoMet: Decarboxylated S-adenosylmethionine
GC7: N1-guanyl-1,7-diaminoheptane
SMOX: Spermine oxidase
DSS: Dextran sodium sulfate
TME: Tumor microenvironm
ROS: Reactive oxygen species
BSAO: Bovine serum amine oxidase
